# Prognostic value of the platelet-to-lymphocyte ratio in lung cancer patients receiving immunotherapy: A systematic review and meta-analysis

**DOI:** 10.1371/journal.pone.0268288

**Published:** 2022-05-06

**Authors:** Haoyu Wang, Cui Li, Ruiyuan Yang, Jing Jin, Dan Liu, Weimin Li

**Affiliations:** 1 Department of Respiratory and Critical Care Medicine, West China Hospital, Sichuan University, Chengdu, Sichuan, China; 2 Institute of Respiratory Health, Frontiers Science Center for Disease-related Molecular Network, West China Hospital, Sichuan University, Chengdu, Sichuan, China; Shuguang Hospital, CHINA

## Abstract

**Background:**

Current studies have revealed that the platelet to lymphocyte ratio (PLR) may lead to a poor prognosis in lung cancer patients receiving immunotherapy. We conducted a meta-analysis to explore the prognostic value of PLR in lung cancer patients receiving immunotherapy.

**Methods:**

We retrieved potential studies from the PubMed, Web of Science, Embase, and Scopus databases up to June 2021 and merged the hazard ratios (HRs) and the corresponding 95% confidence intervals (CIs) to evaluate the association between PLR and overall survival (OS) or progression-free survival.

**Results:**

Fourteen studies involving 1761 patients were included in our meta-analysis. The results indicated that an elevated level of pretreatment PLR was associated with poorer OS and PFS in lung cancer patients receiving immunotherapy (OS: HR = 1.88, 95% CI: 1.37–2.58; PFS: HR = HR = 1.40, 95% CI: 1.11–1.76). The association remained consistent after subgroup analysis and was robust even after sensitivity analysis.

**Conclusions:**

PLR may be a prognostic factor of lung cancer patients receiving immunotherapy, which can lead to worse survival outcomes. However, further studies are necessary for evidence in clinical application.

## Introduction

According to novel cancer statistics, lung cancer remains a common form of cancer whose incidence and mortality rate rank second and first, respectively [1]. Due to advances in diagnostic and therapeutic approaches, the prognosis of lung cancer patients is still worse than that of patients with other types of cancer, with a 5-year relative survival rate less than 20% [1,2].

As the concept of precision medicine develops rapidly, promising types of treatment, including tyrosine kinase inhibitors (TKIs) [3] and immune checkpoint inhibitors (ICIs) [4], have appeared and brought extensive revolution to the therapy for patients with advanced non-small-cell lung cancer (NSCLC) with or without driver gene mutations. Recently, immunotherapy dominated by ICIs targeting programmed death 1 (PD-1), programmed death ligand 1 (PD-L1), and cytotoxic T-lymphocyte-associated protein 4 (CTLA-4) proved to be more effective than traditional chemotherapy and benefitted patients with various kinds of tumors [5,6]. Notwithstanding, only a minority of ICI recipients could obtain a good outcome of survival, while others experienced resistance [7,8], immune-related adverse events (irAEs) [9], or progression [10,11], suggesting the significance of identifying the appropriate population for immunotherapy precisely. To date, several biomarkers based on tissue samples have been applied in clinical practice, such as PD-L1 expression [12] and tumor mutational burden (TMB) [13]. Nevertheless, these techniques are expensive and invasive and fail to display the roles of predictors for ICI response and prognosis in NSCLC patients [14,15]. Instead, some serum biomarkers, such as neutrophil to lymphocyte ratio (NLR), platelet to lymphocyte ratio (PLR), Glasgow Prognostic Score (GPS), and modified Glasgow Prognostic Score (mGPS), which are easy to obtain from routine clinical methods, showed good prognostic significance in NSCLC patients [16–18]. However, whether they can help identify immunotherapy recipients has been poorly studied. Thus, we aimed to explore the prognostic value of the PLR in immunotherapy recipients with lung cancer by conducting a meta-analysis and hope to redound on clinical determination.

## Methods

### Ethics statement

All procedures performed in studies that involved human participants were in accordance with the ethical standards of the institutional and/or national research committee and with the 1964 Helsinki Declaration and its later amendments or comparable ethical standards. For this type of study, formal consent is not needed.

### Protocol and registration

The present meta-analysis was reported in accordance with the Preferred Reporting Items for Systematic Review and Meta-Analyses (PRISMA) statement [19] and was registered at the International Prospective Register of Systematic Reviews (PROSPERO): number CRD42021258295.

### Search strategy

The systematic search of the literature was performed in the PubMed, Web of Science, Scopus, and Embase databases up to June 2021 without restriction for publication years. The following words were used to evaluate the association between PLR and survival in lung cancer patients receiving immunotherapy: “pulmonary neoplasms”, “lung cancer”, “immunotherapy”, “immune checkpoint inhibitor”, “programmed death 1”, and “platelet-to-lymphocyte ratio”. Additional articles were manually retrieved from the reference lists of relevant articles, and the included articles were restricted to English. The detailed search strategy for PubMed is presented in **S1 File**.

### Eligibility criteria

Studies were included if they met the following criteria: 1) all patients were pathologically diagnosed with lung cancer and received immunotherapy; 2) studies investigated the prognostic value of PLR; 3) the outcomes included the OS or PFS with hazard ratios (HRs) and corresponding 95% confidence intervals (95% CIs); 4) retrospective or prospective studies with the full-text paper published before June 2021; and 5) the latest study was included if several studies had an overlapping population.

Studies were excluded if they met the following criteria: 1) reviews, conference abstracts, case reports, letters, or comments; 2) laboratory studies of clinical samples, cell lines, or animals; 3) insufficient data of PLR or lack of control; 4) full-text paper written in English was not available.

### Data extraction

Two researchers independently extracted the following data from the eligible studies: family name of the first author, year of publication, study design, ethnicity, follow-up (months), sample size, type of immune checkpoint inhibitor, detection time, PLR cutoff value, and outcomes with HRs and their corresponding 95% CIs. Any disagreement was resolved by discussion and consensus.

### Risk of bias assessment

The risk of bias of each study included was assessed by the Newcastle–Ottawa quality assessment Scale (NOS), and studies labeled with 6 points or higher were regarded as high-quality studies [20].

### Statistical analysis

Statistical analysis was performed via R (version 4.0.3) and R Studio (version 1.3.1). HRs from the multivariate analysis were used wherever available, and HRs from univariate analysis were substitutes if only the univariate analysis was performed. In addition, HRs were estimated by applying the Tierney method if they were not provided directly [21]. Pooled HRs and 95% CIs were combined with the random effects or fixed effects model according to the heterogeneity. Heterogeneity was assessed by forest plots, Q tests, and I^2^ statistics. Significant heterogeneity was defined as a p value < 0.05 and I^2^ > 50%, and the random effects model was used. Otherwise, we chose the fixed effects model. Subgroup analyses were performed to investigate potential confounding factors of this meta-analysis. Sensitivity analysis was conducted by excluding each study independently from our meta-analysis to determine the overrepresentation of every single study. Publication bias was evaluated by Begg’s test and funnel plots. A P value < 0.05 was considered statistically significant.

## Results

### Literature search and risk of bias assessment

**Fig 1:**
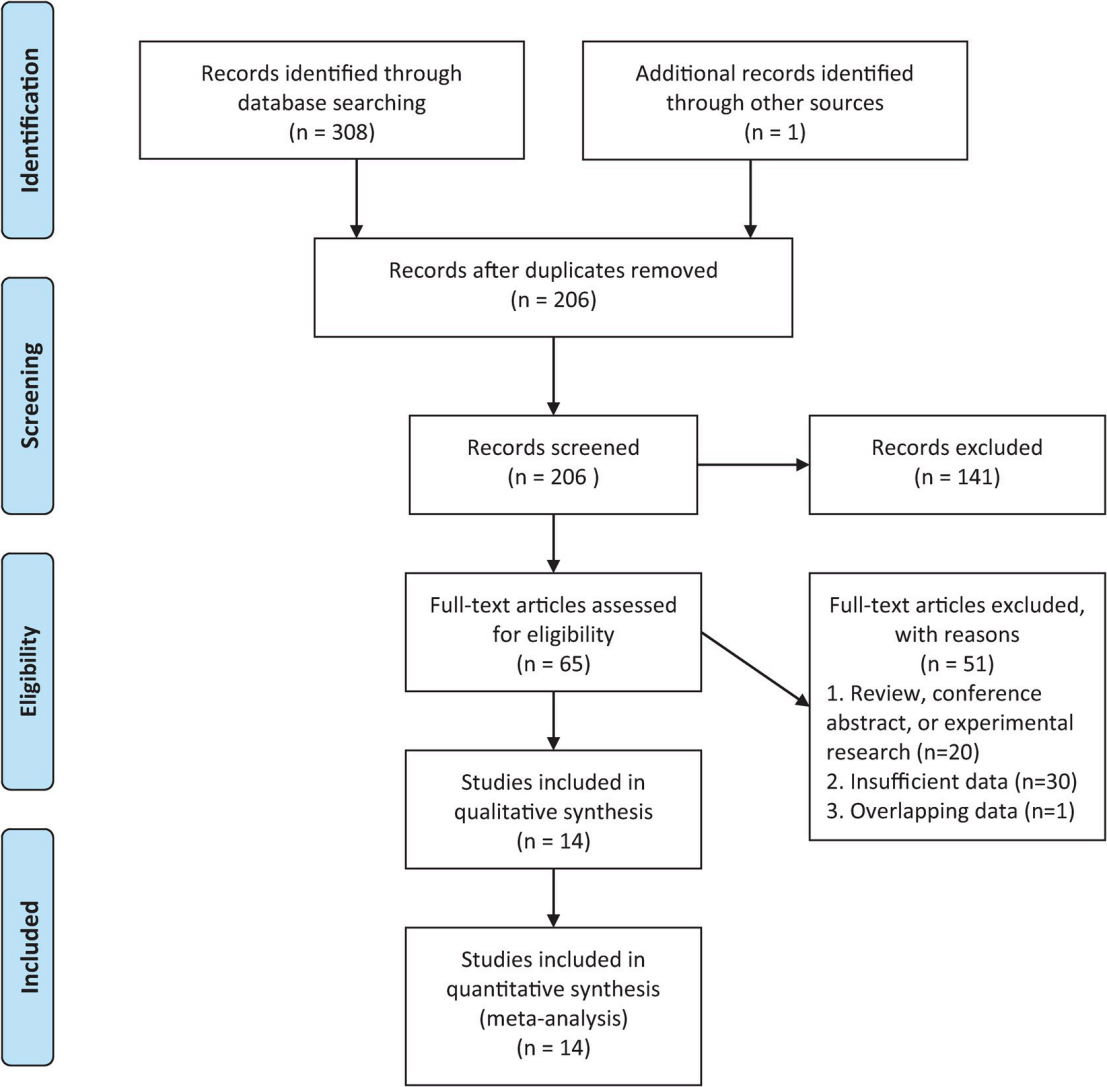
PRISMA flow diagram of the literature search in this meta-analysis.

The PRISMA flow diagram and checklist of this meta-analysis are presented in **Fig 1** and the **S1 Checklist**. A total of 309 separate publications were initially retrieved through our search strategy, and 206 articles remained after removing duplicates. We found 65 potentially eligible studies according to titles and abstracts and then screened the full-text versions of them. Finally, 14 studies were included in this meta-analysis. The NOS scores varied from 6 to 9, which demonstrated a low risk of bias in these studies.

### Characteristics of the included studies

The main characteristics of all 14 studies that met our inclusion and exclusion criteria are displayed in **Table 1** [22–35]. All 14 studies were retrospective, and 7 studies enrolled Asian patients, while 7 studies enrolled European patients. The sample size ranged from 24 to 404, with a sum of 1761. Most studies only analyzed the pretreatment PLR, and only 4 studies evaluated the results of posttreatment PLR. All studies defined OS as the time from inclusion to the date of death or last follow-up, and PFS was defined as the time from the initial date of immunotherapy to the date of progression or death.

**Table 1:** Main characteristics of the studies included.

Source	Design	Ethnicity	MFP (months)	Sample size	ICI	Detection time	Cutoff value	Outcome	NOS
Diem 2017	RO	European	NA	52	N	Pre	262	OS	6
Suh 2017	RO	Asian	26.2	54	N, P	Pre, Post	169	OS, PFS	8
Svaton 2018	RO	European	NA	120	N	Pre	169.1	OS, PFS	7
Takeda 2018	RO	Asian	NA	30	N	Pre, Post	150	PFS	6
Dusselier 2019	RO	European	NA	59	N	Pre, Post	262	OS	8
Liu 2019	RO	Asian	6.9	44	N	Pre	144	OS, PFS	6
Pavan 2019	RO	European	56.3	184	N, P, A	Pre	180	OS, PFS	8
Jiang 2020	RO	Asian	7.1	76	N, D	Pre, Post	168.13	OS, PFS	6
Katayama2020	RO	Asian	NA	81	A	Pre	262	OS, PFS	6
Matsubara 2020	RO	Asian	NA	24	A	Pre	150	OS	8
Russo 2020	RO	European	NA	187	N	Pre	200	OS, PFS	7
Takada 2020	RO	Asian	13.8	226	N, P	Pre	245	OS, PFS	7
Ksienski 2021	RO	European	9.2	220	P	Pre	441.8	OS	8
Lobefaro 2021	RO	European	29.0	404	NA	Pre	255	OS, PFS	9

**Abbreviation:** MFP: Median follow-up; ICI: Immune checkpoint inhibitor; NOS: Newcastle–Ottawa quality assessment Scale; RO: Retrospective study; NA: Not available; N: Nivolumab; P: Pembrolizumab; A: Atezolizumab; D: Durvalumab; Pre: Pretreatment; Post: Posttreatment; OS: Overall survival; PFS: Progression-free survival.

### Impact of the PLR on OS and PFS

**Fig 2:**
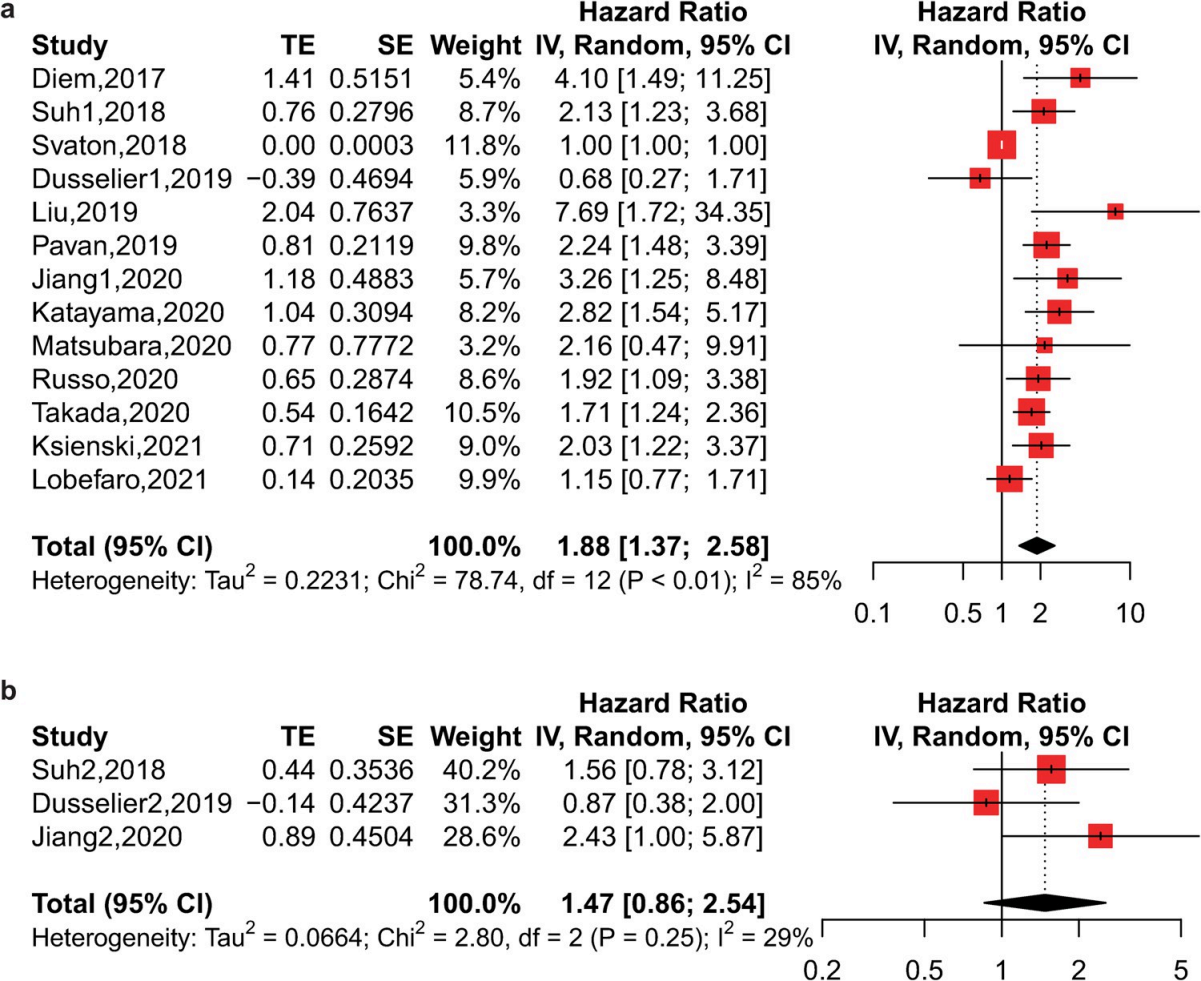
Forest plot of the association between PLR and OS of lung cancer patients receiving immunotherapy. a) Forest plot of pretreatment PLR; b) Forest plot of posttreatment PLR.

**Fig 3:**
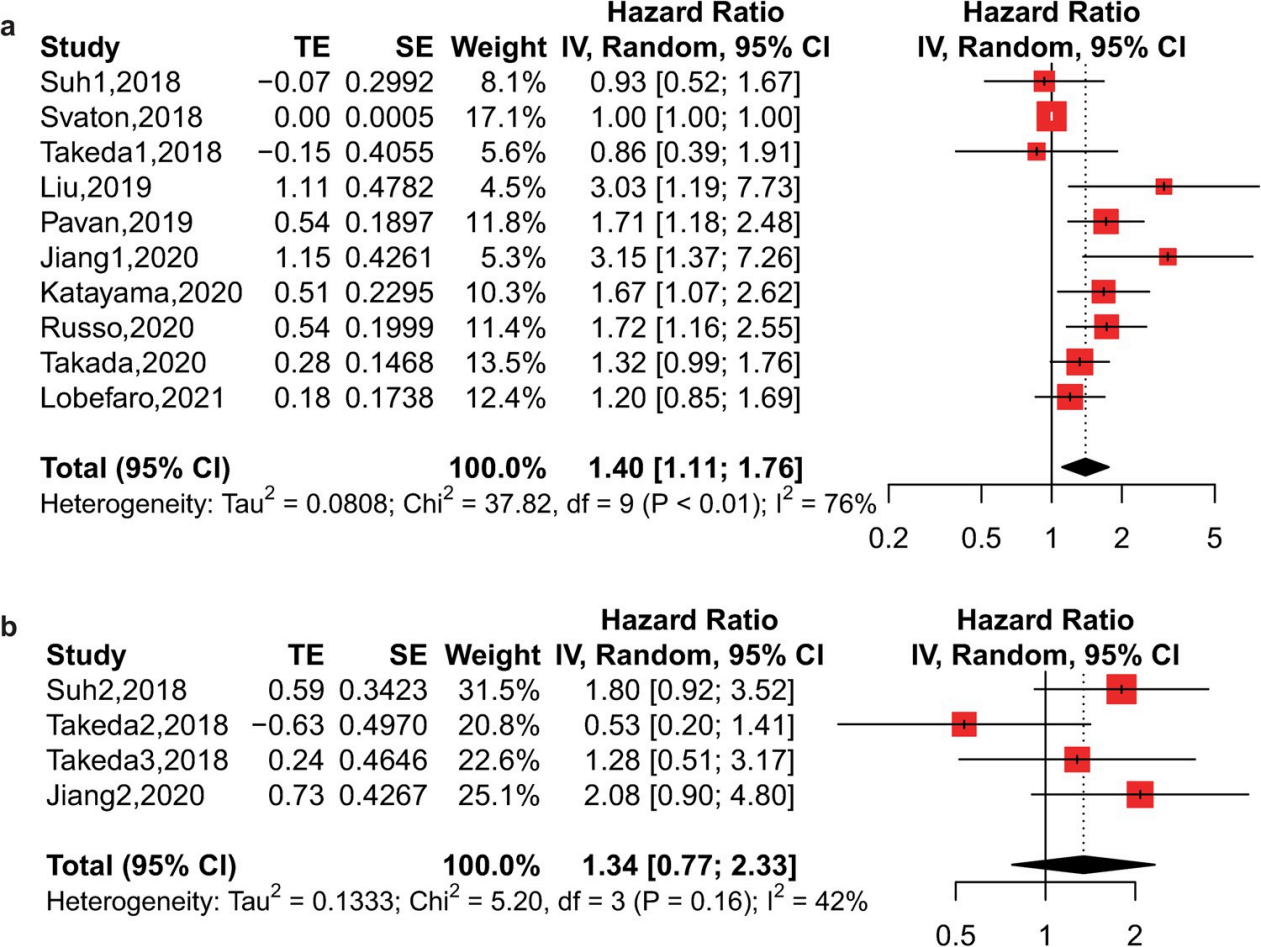
Forest plot of the association between PLR and PFS of lung cancer patients receiving immunotherapy. a) Forest plot of pretreatment PLR; b) Forest plot of posttreatment PLR.

A total of 13 studies on 1731 patients receiving immunotherapy contributed to the primary meta-analysis. From the pooled analysis of PLR and OS, we found that a higher pretreatment PLR was associated with poorer OS with high heterogeneity (HR = 1.88, 95% CI: 1.37–2.58, p<0.01, I^2^ = 85%, p<0.01) (**Fig 2A**). However, the posttreatment PLR did not seem to be correlated with OS, with small data from only 3 studies (HR = 1.47, 95% CI: 0.86–2.54, p = 0.16, I^2^ = 29%, p = 0.25) (**Fig 2B**). For PFS, 10 studies with 1406 patients were analyzed, and we found that a higher pretreatment PLR was also associated with worse PFS with high heterogeneity (HR = 1.40, 95% CI: 1.11–1.76, p<0.01, I^2^ = 76%, p<0.01) (**Fig 3A**). Additionally, the posttreatment PLR was not related to PFS from the pooled analysis of only 4 studies (HR = 1.34, 95% CI: 0.77–2.33, p = 0.30, I^2^ = 42%, p = 0.16) (**Fig 3B**).

### Subgroup analysis

To detect the potential origins of the heterogeneity among the included studies, we then conducted subgroup analyses according to ethnicity, sample size, median follow-up (months), and cutoff value. As displayed in **Table 2**, the OS and PFS for most subgroups showed a similar trend to the primary analysis. However, the pretreatment PLR was not related to PFS in the European, small sample size (<100), and low cutoff value (<169) subgroups. Interestingly, the pretreatment PLR was significantly unrelated to both OS and PFS in studies whose median follow-up was unavailable (HR = 1.69, 95% CI: 0.98–2.92, p = 0.06, I^2^ = 80%, p<0.01). Surprisingly, the pooled HR of the higher cutoff value subgroup appeared to be less than that of the subgroup with a lower cutoff value (OS: 1.71 to 3.62, PFS: 1.31 to 1.98). Regardless, the differences were not statistically significant (OS: p = 0.06, PFS: p = 0.36), and the heterogeneity of the high cutoff value subgroup was high (I^2^ = 86%, p<0.01).

**Table 2:** Results of subgroup analysis.

	N	OS				N	PFS			
		Association		Heterogeneity			Association		Heterogeneity	
		HR (95%CI)	p	I^2^	p		HR (95%CI)	p	I^2^	p
**Ethnicity**										
Asian	6	2.23 (1.65, 3.00)	<0.01	19%	0.29	6	1.48 (1.06, 2.08)	0.02	51%	0.07
European	7	1.54 (1.06, 2.25)	0.02	83%	<0.01	4	1.32 (0.97, 1.81)	0.08	82%	<0.01
**Sample size**										
<100	7	2.44 (1.52, 3.91)	<0.01	48%	0.07	5	1.58 (0.98, 2.56)	0.06	59%	0.04
≥100	6	1.56 (1.10, 2.21)	0.01	87%	<0.01	5	1.31 (1.02, 1.69)	0.03	80%	<0.01
**Median follow-up (months)**										
<12	3	2.84 (1.51, 5.36)	<0.01	36%	0.21	2	3.10 (1.66, 5.78)	<0.01	0%	0.95
≥12	4	1.72 (1.28, 2.30)	<0.01	50%	0.11	4	1.32 (1.08, 1.61)	<0.01	15%	0.32
NA	6	1.69 (0.98, 2.92)	0.06	80%	<0.01	4	1.27 (0.88, 1.84)	0.20	76%	<0.01
**Cutoff value**										
<169	3	3.62 (1.77, 7.37)	<0.01	0%	0.48	3	1.98 (0.83, 4.70)	0.122	68%	0.046
≥169	10	1.71 (1.24, 2.36)	<0.01	86%	<0.01	7	1.31 (1.05, 1.64)	0.018	76%	<0.01
**Overall**	13	1.88 (1.37, 2.58)	<0.01	85%	<0.01	10	1.40 (1.11, 1.76)	<0.01	76%	<0.01

**Abbreviation:** N: Number of studies; OS: Overall survival; PFS: Progression-free survival; HR: Hazard ratio; CI: Confidence interval.

### Sensitivity analysis

**Fig 4:**
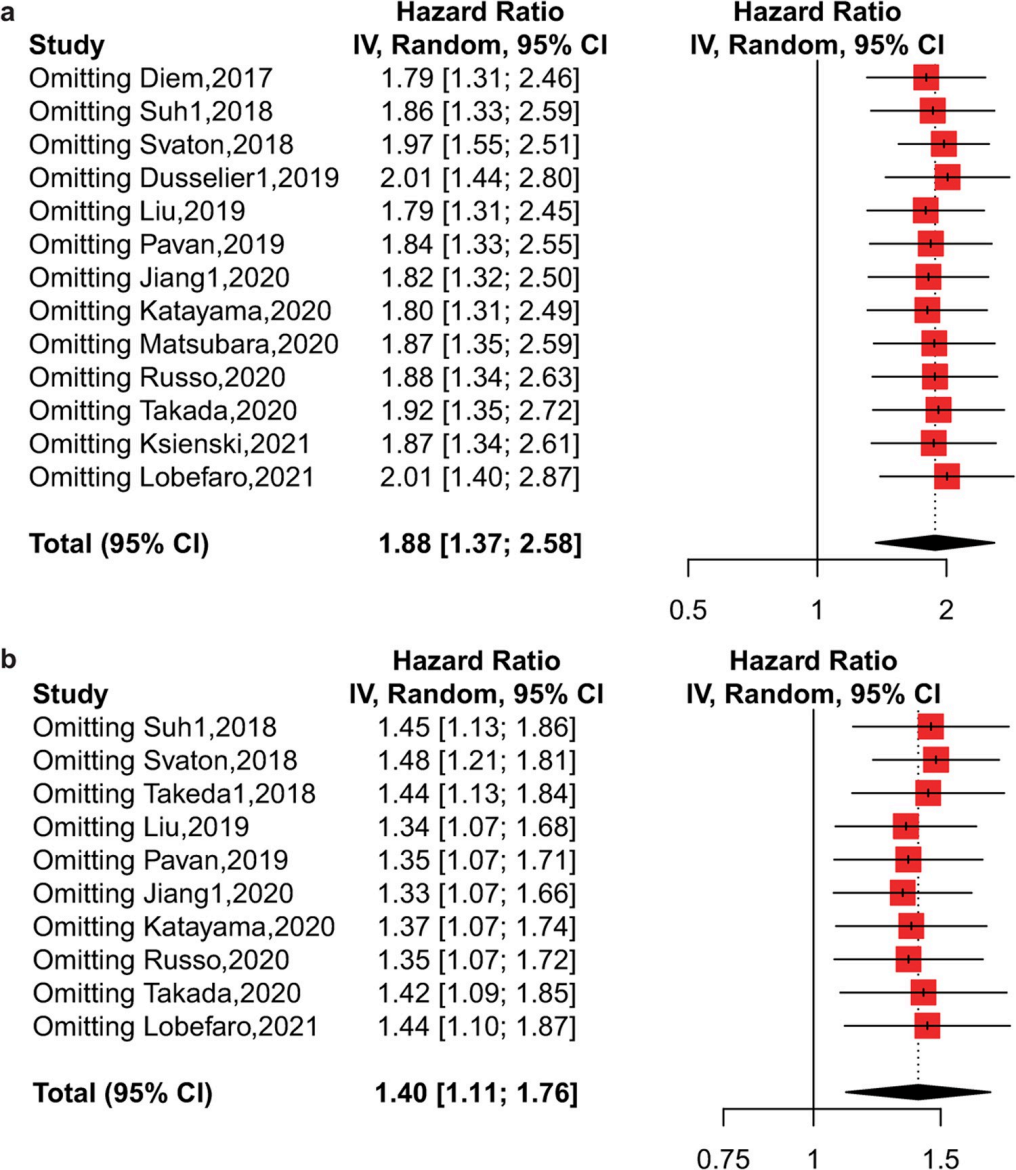
Sensitivity analysis by excluding each study from the meta-analysis. a) Sensitivity analysis for OS; b) sensitivity analysis for PFS.

Subsequently, we conducted a sensitivity analysis to further explore the potential cause of heterogeneity for OS and PFS (**Fig 4A and 4B**). As shown, the pooled HRs and corresponding 95% CIs were robust in our meta-analysis. Nevertheless, when excluding the study of Svaton [33], the heterogeneity was significantly reduced for both OS (I^2^ = 46%, p = 0.04) and PFS (I^2^ = 38%, p = 0.11), suggesting that this study might be the main source of heterogeneity. Therefore, we conducted an additional analysis for OS and PFS after removing the study of Svaton, and the results showed that elevated pretreatment PLR was still associated with poor OS (HR = 1.97, 95% CI: 1.55–2.51, p<0.01) and PFS (HR = 1.45, 95% CI: 1.25–1.68, p<0.01).

### Publication bias

**Fig 5:**
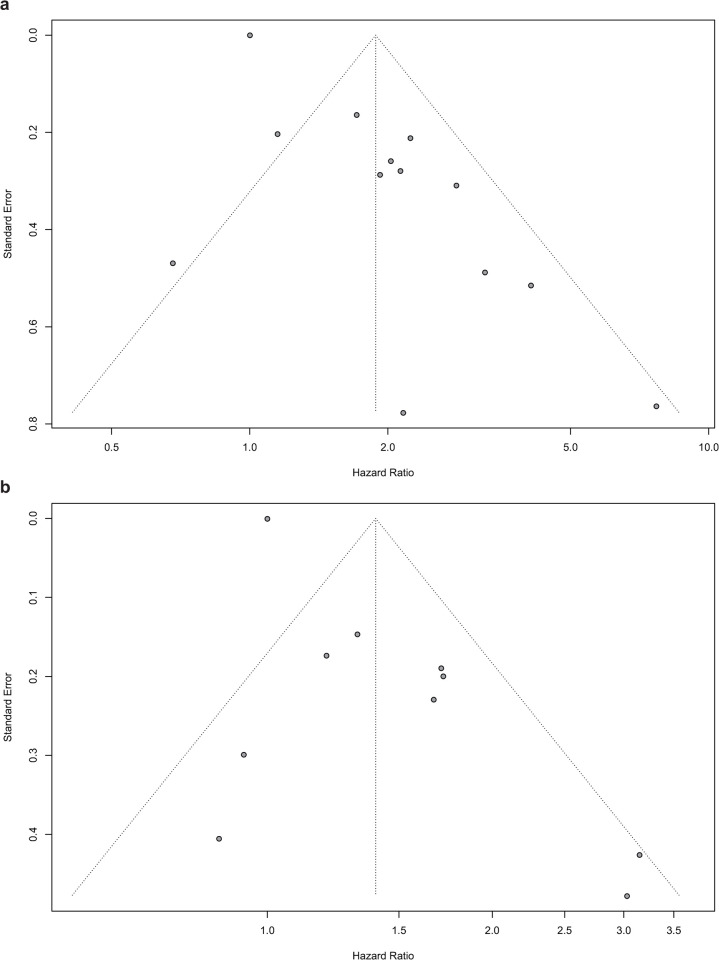
Funnel plots for detecting publication bias. a) funnel plot for OS; b) funnel plots for PFS.

Funnel plots and Begg’s test were applied to assess publication bias. The funnel plots for OS and PFS were basically symmetrical (**Fig 5A and 5B**), and the results of Begg’s test showed that there was no significant publication bias (OS: p = 0.81, PFS: p = 0.65).

## Discussion

The results of our meta-analysis demonstrated the prognostic value of the PLR in lung cancer patients receiving immunotherapy. From a total of 14 studies on 1761 patients, we found that an elevated pretreatment PLR was significantly associated with poorer OS and PFS in these patients. However, the posttreatment PLR was unrelated to either OS or PFS, but the results were from only 3 and 4 studies, respectively. Furthermore, pretreatment PLR may have a worse effect on Asian patients than on European patients. Lung cancer remains the main cause of cancer death globally [1], and recently, immunotherapy has been a novel approach for cancer treatment through targets, such as PD-1, PD-L1, and CTLA-4, to enhance the patients’ immune system [5,6]. Immunotherapy proved to be effective in many types of cancer [36,37]; however, not all patients can benefit from immunotherapy. Previous studies demonstrated that PD-L1 expression level tests in tissue samples [38], tumor mutation burden (TMB) [39], and microsatellite instability (MSI) [40] might be predictive or prognostic factors for immunotherapy. However, these examinations are either expensive or invasive, and some patients cannot benefit from these examinations, perhaps due to differences in immune status among patients [41]. Therefore, a cheaper and noninvasive method for predicting the response and survival of immunotherapy is necessary. Systematic inflammation has always been considered to contribute to tumorigenesis and immune abnormalities [42,43], and several inflammation biomarkers have been proven to be prognostic factors for lung cancer and cancer patients receiving immunotherapy, such as NLR, GPS, and mGPS [16–18]. Two previous meta-analyses have already focused on and confirmed the association between PLR and lung cancer prognosis, but they did not include any study about immunotherapy recipients, and they did not discuss the posttreatment PLR [44,45]. Therefore, we focused on PLR and aimed to explore the association between PLR and the survival of lung cancer patients receiving immunotherapy.

Previous studies demonstrated that the lung plays a crucial role in the biogenesis of platelets and can produce nearly 50% of them, suggesting that the interaction between the lung and platelets may be an important process in the microenvironment of the lung [46], and high pretreatment counts proved to be risk factors for venous thromboembolism in lung cancer patients, which may lead to a worse outcome of survival [47]. Moreover, platelets play a vital role in the lung microenvironment. Novel evidence has proposed that platelets are able to educate tumor cells by structural component transference and special RNA splicing in tumor-associated signals, which can lead to the altered adverse phenotype of higher proliferation, epithelial-mesenchymal transition (EMT), and stem-like features [48,49]. As the techniques of liquid biopsy are burgeoning, the detection of these tumor-educated platelets (TEPs) may be useful in cancer diagnosis and prognosis prediction [50].

Two previous meta-analyses also evaluated the prognostic value of PLR and showed a similar trend to ours. However, the first study by Xu enrolled various kinds of cancers, and only 8 studies were about lung cancer [51]. In addition, their eligibility criteria were not strictly limited, and many conference abstracts were enrolled, which may lead to the risk of bias and lack of information for the study design and other details. The second study by Zhang included only 8 studies on 686 patients, and their analysis mixed the pretreatment and posttreatment PLR, which may lead to overlapping data [52]. Different from the former studies, we constructed stricter eligibility criteria to avoid the risk of bias, included more research articles to increase the robustness of our results, and analyzed the pretreatment and posttreatment PLR to avoid duplication. Moreover, we revealed that the sample size and cutoff value might affect the prognostic value of PLR, and the HRs of PFS in Asian patients might be higher than those in European patients. We hypothesized that the discrepancy in the immune status among ethnicities led to it. Additionally, the posttreatment PLR seemed not to be associated with either OS or PFS, which may be due to the small sample of articles, so the results should be applied carefully in clinical practice. Since blood parameters are dynamically changing, follow-up is especially important.

The current study has some limitations. First, the heterogeneity among our included studies was high, and when excluding the study of Svaton, the heterogeneity was obviously reduced; thus, we regarded this research as a potential source of heterogeneity, although it had no significant bias after our reassessment. Second, all included studies were retrospective, and some articles did not provide the HRs and corresponding 95% CIs directly, which may attenuate the robustness of our results. Third, the type of immune checkpoint inhibitor varied greatly among the included studies, but we could not conduct a subgroup analysis due to a lack of data.

## Conclusion

Generally, the present meta-analysis demonstrated that high pretreatment PLR is a prognostic factor for lung cancer patients receiving immunotherapy. As a cheap, noninvasive, and easily available biomarker, pretreatment PLR can help clinicians make early identification of the immunotherapy recipients’ benefit and prognosis. Moreover, future therapeutic approaches targeting platelets may also contribute to the efficiency of immunotherapy given that platelets also play a critical role in tumors. However, given the limitations, the results should be applied with caution, especially in clinical practice. Moreover, more prospective cohort studies with large samples and posttreatment follow-up of PLR are needed to confirm our results, and studies regarding platelets in the tumor microenvironment are also needed.

## Supporting information

S1 Checklist PRISMA checklist of the meta-analysis. (DOC)

S1 Fig The forest plot of the association between pretreatment PLR and the survival of lung cancer patients receiving immunotherapy after revoing the potential source of heterogeneity. a) forest plot for OS; b) forest plot for PFS.
(TIF)

S1 File Search strategy for meta-analysis (PubMed via NLM). (DOCX)

S2 File The minimal dataset necessary to replicate our findings. (XLSX)

## References

[1] Sung H, Ferlay J, Siegel RL, Laversanne M, Soerjomataram I, Jemal A, et al. Global Cancer Statistics 2020: GLOBOCAN Estimates of Incidence and Mortality Worldwide for 36 Cancers in 185 Countries. CA: a cancer journal for clinicians. 2021;71(3):209–49. Epub 2021/02/05. doi: 10.3322/caac.21660 . 33538338

[2] Siegel RL, Miller KD, Fuchs HE, Jemal A. Cancer Statistics, 2021. CA: a cancer journal for clinicians. 2021;71(1):7–33. Epub 2021/01/13. doi: 10.3322/caac.21654 . 33433946

[3] Mok TS, Wu YL, Thongprasert S, Yang CH, Chu DT, Saijo N, et al. Gefitinib or carboplatin-paclitaxel in pulmonary adenocarcinoma. The New England journal of medicine. 2009;361(10):947–57. Epub 2009/08/21. doi: 10.1056/NEJMoa0810699 . 19692680

[4] Herbst RS, Baas P, Kim DW, Felip E, Pérez-Gracia JL, Han JY, et al. Pembrolizumab versus docetaxel for previously treated, PD-L1-positive, advanced non-small-cell lung cancer (KEYNOTE-010): a randomised controlled trial. Lancet (London, England). 2016;387(10027):1540–50. Epub 2015/12/30. doi: 10.1016/S0140-6736(15)01281-7 . 26712084

[5] Keir ME, Butte MJ, Freeman GJ, Sharpe AH. PD-1 and its ligands in tolerance and immunity. Annual review of immunology. 2008;26:677–704. Epub 2008/01/05. doi: 10.1146/annurev.immunol.26.021607.090331 . 18173375 PMC10637733

[6] Postow MA, Callahan MK, Wolchok JD. Immune Checkpoint Blockade in Cancer Therapy. Journal of clinical oncology: official journal of the American Society of Clinical Oncology. 2015;33(17):1974–82. Epub 2015/01/22. doi: 10.1200/JCO.2014.59.4358 . 25605845 PMC4980573

[7] Bagchi S, Yuan R, Engleman EG. Immune Checkpoint Inhibitors for the Treatment of Cancer: Clinical Impact and Mechanisms of Response and Resistance. Annual review of pathology. 2021;16:223–49. Epub 2020/11/17. doi: 10.1146/annurev-pathol-042020-042741 . 33197221

[8] Conforti F, Pala L, Bagnardi V, De Pas T, Martinetti M, Viale G, et al. Cancer immunotherapy efficacy and patients’ sex: a systematic review and meta-analysis. The Lancet Oncology. 2018;19(6):737–46. Epub 2018/05/21. doi: 10.1016/S1470-2045(18)30261-4 . 29778737

[9] Baxi S, Yang A, Gennarelli RL, Khan N, Wang Z, Boyce L, et al. Immune-related adverse events for anti-PD-1 and anti-PD-L1 drugs: systematic review and meta-analysis. BMJ (Clinical research ed). 2018;360:k793. Epub 2018/03/16. doi: 10.1136/bmj.k793 . 29540345 PMC5851471

[10] Billan S, Kaidar-Person O, Gil Z. Treatment after progression in the era of immunotherapy. The Lancet Oncology. 2020;21(10):e463–e76. Epub 2020/10/02. doi: 10.1016/S1470-2045(20)30328-4 . 33002442

[11] Remon J, Passiglia F, Ahn MJ, Barlesi F, Forde PM, Garon EB, et al. Immune Checkpoint Inhibitors in Thoracic Malignancies: Review of the Existing Evidence by an IASLC Expert Panel and Recommendations. Journal of thoracic oncology: official publication of the International Association for the Study of Lung Cancer. 2020;15(6):914–47. Epub 2020/03/18. doi: 10.1016/j.jtho.2020.03.006 . 32179179

[12] Ilie M, Long-Mira E, Bence C, Butori C, Lassalle S, Bouhlel L, et al. Comparative study of the PD-L1 status between surgically resected specimens and matched biopsies of NSCLC patients reveal major discordances: a potential issue for anti-PD-L1 therapeutic strategies. Annals of oncology: official journal of the European Society for Medical Oncology. 2016;27(1):147–53. Epub 2015/10/21. doi: 10.1093/annonc/mdv489 . 26483045

[13] Yarchoan M, Hopkins A, Jaffee EM. Tumor Mutational Burden and Response Rate to PD-1 Inhibition. The New England journal of medicine. 2017;377(25):2500–1. Epub 2017/12/21. doi: 10.1056/NEJMc1713444 . 29262275 PMC6549688

[14] Borghaei H, Paz-Ares L, Horn L, Spigel DR, Steins M, Ready NE, et al. Nivolumab versus Docetaxel in Advanced Nonsquamous Non-Small-Cell Lung Cancer. The New England journal of medicine. 2015;373(17):1627–39. Epub 2015/09/29. doi: 10.1056/NEJMoa1507643 . 26412456 PMC5705936

[15] Hellmann MD, Ciuleanu TE, Pluzanski A, Lee JS, Otterson GA, Audigier-Valette C, et al. Nivolumab plus Ipilimumab in Lung Cancer with a High Tumor Mutational Burden. The New England journal of medicine. 2018;378(22):2093–104. Epub 2018/04/17. doi: 10.1056/NEJMoa1801946 . 29658845 PMC7193684

[16] Jin J, Hu K, Zhou Y, Li W. Clinical utility of the modified Glasgow prognostic score in lung cancer: A meta-analysis. PloS one. 2017;12(9):e0184412. Epub 2017/09/09. doi: 10.1371/journal.pone.0184412 . 28886134 PMC5590927

[17] Leung EY, Scott HR, McMillan DC. Clinical utility of the pretreatment glasgow prognostic score in patients with advanced inoperable non-small cell lung cancer. Journal of thoracic oncology: official publication of the International Association for the Study of Lung Cancer. 2012;7(4):655–62. Epub 2012/03/20. doi: 10.1097/JTO.0b013e318244ffe1 . 22425914

[18] Mandaliya H, Jones M, Oldmeadow C, Nordman, II. Prognostic biomarkers in stage IV non-small cell lung cancer (NSCLC): neutrophil to lymphocyte ratio (NLR), lymphocyte to monocyte ratio (LMR), platelet to lymphocyte ratio (PLR) and advanced lung cancer inflammation index (ALI). Translational lung cancer research. 2019;8(6):886–94. Epub 2020/02/06. doi: 10.21037/tlcr.2019.11.16 . 32010567 PMC6976360

[19] Liberati A, Altman DG, Tetzlaff J, Mulrow C, Gøtzsche PC, Ioannidis JP, et al. The PRISMA statement for reporting systematic reviews and meta-analyses of studies that evaluate health care interventions: explanation and elaboration. PLoS medicine. 2009;6(7):e1000100. Epub 2009/07/22. doi: 10.1371/journal.pmed.1000100 . 19621070 PMC2707010

[20] Wells BS DOC GA, Peterson J, Welch V, Losos M, Tugwell P. The Newcastle-Ottawa Scale (NOS) for assessing the quality of nonrandomised studies in meta-analyses 2013. Available from: http://www.ohri.ca/programs/clinical_epidemiology/oxford.asp.

[21] Tierney JF, Stewart LA, Ghersi D, Burdett S, Sydes MR. Practical methods for incorporating summary time-to-event data into meta-analysis. Trials. 2007;8:16. Epub 2007/06/09. doi: 10.1186/1745-6215-8-16 . 17555582 PMC1920534

[22] Diem S, Schmid S, Krapf M, Flatz L, Born D, Jochum W, et al. Neutrophil-to-Lymphocyte ratio (NLR) and Platelet-to-Lymphocyte ratio (PLR) as prognostic markers in patients with non-small cell lung cancer (NSCLC) treated with nivolumab. Lung Cancer. 2017;111:176–81. Epub 2017/08/26. doi: 10.1016/j.lungcan.2017.07.024 . 28838390

[23] Dusselier M, Deluche E, Delacourt N, Ballouhey J, Egenod T, Melloni B, et al. Neutrophil-to-lymphocyte ratio evolution is an independent predictor of early progression of second-line nivolumab-treated patients with advanced non-small-cell lung cancers. PloS one. 2019;14(7):e0219060. Epub 2019/07/18. doi: 10.1371/journal.pone.0219060 . 31314761 PMC6636729

[24] Jiang M, Peng W, Pu X, Chen B, Li J, Xu F, et al. Peripheral Blood Biomarkers Associated With Outcome in Non-small Cell Lung Cancer Patients Treated With Nivolumab and Durvalumab Monotherapy. Front Oncol. 2020;10:913. Epub 2020/07/23. doi: 10.3389/fonc.2020.00913 . 32695663 PMC7339928

[25] Katayama Y, Yamada T, Chihara Y, Tanaka S, Tanimura K, Okura N, et al. Significance of inflammatory indexes in atezolizumab monotherapy outcomes in previously treated non-small-cell lung cancer patients. Sci Rep. 2020;10(1):17495. Epub 2020/10/17. doi: 10.1038/s41598-020-74573-0 . 33060826 PMC7566597

[26] Ksienski D, Wai ES, Alex D, Croteau NS, Freeman AT, Chan A, et al. Prognostic significance of the neutrophil-to-lymphocyte ratio and platelet-to-lymphocyte ratio for advanced non-small cell lung cancer patients with high PD-L1 tumor expression receiving pembrolizumab. Translational lung cancer research. 2021;10(1):355–67. Epub 2021/02/12. doi: 10.21037/tlcr-20-541 . 33569318 PMC7867765

[27] Liu J, Li S, Zhang S, Liu Y, Ma L, Zhu J, et al. Systemic immune-inflammation index, neutrophil-to-lymphocyte ratio, platelet-to-lymphocyte ratio can predict clinical outcomes in patients with metastatic non-small-cell lung cancer treated with nivolumab. J Clin Lab Anal. 2019;33(8):e22964. Epub 2019/07/10. doi: 10.1002/jcla.22964 . 31282096 PMC6805305

[28] Lobefaro R, Viscardi G, Di Liello R, Massa G, Iacovino ML, Sparano F, et al. Immunotherapy in advanced Non-Small Cell Lung Cancer patients with poor performance status: The role of clinical-pathological variables and inflammatory biomarkers. Lung Cancer. 2021;152:165–73. Epub 2021/01/10. doi: 10.1016/j.lungcan.2020.12.027 . 33421923

[29] Matsubara T, Takamori S, Haratake N, Toyozawa R, Miura N, Shimokawa M, et al. The impact of immune-inflammation-nutritional parameters on the prognosis of non-small cell lung cancer patients treated with atezolizumab. J Thorac Dis. 2020;12(4):1520–8. Epub 2020/05/13. doi: 10.21037/jtd.2020.02.27 . 32395289 PMC7212122

[30] Pavan A, Calvetti L, Dal Maso A, Attili I, Del Bianco P, Pasello G, et al. Peripheral Blood Markers Identify Risk of Immune-Related Toxicity in Advanced Non-Small Cell Lung Cancer Treated with Immune-Checkpoint Inhibitors. Oncologist. 2019;24(8):1128–36. Epub 2019/04/25. doi: 10.1634/theoncologist.2018-0563 . 31015312 PMC6693718

[31] Russo A, Russano M, Franchina T, Migliorino MR, Aprile G, Mansueto G, et al. Neutrophil-to-Lymphocyte Ratio (NLR), Platelet-to-Lymphocyte Ratio (PLR), and Outcomes with Nivolumab in Pretreated Non-Small Cell Lung Cancer (NSCLC): A Large Retrospective Multicenter Study. Adv Ther. 2020;37(3):1145–55. Epub 2020/02/01. doi: 10.1007/s12325-020-01229-w . 32002809

[32] Suh KJ, Kim SH, Kim YJ, Kim M, Keam B, Kim TM, et al. Post-treatment neutrophil-to-lymphocyte ratio at week 6 is prognostic in patients with advanced non-small cell lung cancers treated with anti-PD-1 antibody. Cancer Immunol Immunother. 2018;67(3):459–70. Epub 2017/12/06. doi: 10.1007/s00262-017-2092-x . 29204702 PMC11028357

[33] Svaton M, Zemanova M, Skrickova J, Jakubikova L, Kolek V, Kultan J, et al. Chronic Inflammation as a Potential Predictive Factor of Nivolumab Therapy in Non-small Cell Lung Cancer. Anticancer Res. 2018;38(12):6771–82. Epub 2018/12/07. doi: 10.21873/anticanres.13048 . 30504389

[34] Takada K, Takamori S, Yoneshima Y, Tanaka K, Okamoto I, Shimokawa M, et al. Serum markers associated with treatment response and survival in non-small cell lung cancer patients treated with anti-PD-1 therapy. Lung Cancer. 2020;145:18–26. Epub 2020/05/11. doi: 10.1016/j.lungcan.2020.04.034 . 32388276

[35] Takeda T, Takeuchi M, Saitoh M, Takeda S. Neutrophil-to-lymphocyte ratio after four weeks of nivolumab administration as a predictive marker in patients with pretreated non-small-cell lung cancer. Thorac Cancer. 2018;9(10):1291–9. Epub 2018/08/21. doi: 10.1111/1759-7714.12838 . 30126063 PMC6166075

[36] Brahmer J, Reckamp KL, Baas P, Crinò L, Eberhardt WE, Poddubskaya E, et al. Nivolumab versus Docetaxel in Advanced Squamous-Cell Non-Small-Cell Lung Cancer. The New England journal of medicine. 2015;373(2):123–35. Epub 2015/06/02. doi: 10.1056/NEJMoa1504627 . 26028407 PMC4681400

[37] Massard C, Gordon MS, Sharma S, Rafii S, Wainberg ZA, Luke J, et al. Safety and Efficacy of Durvalumab (MEDI4736), an Anti-Programmed Cell Death Ligand-1 Immune Checkpoint Inhibitor, in Patients With Advanced Urothelial Bladder Cancer. Journal of clinical oncology: official journal of the American Society of Clinical Oncology. 2016;34(26):3119–25. Epub 2016/06/09. doi: 10.1200/JCO.2016.67.9761 . 27269937 PMC5569690

[38] Fusi A, Festino L, Botti G, Masucci G, Melero I, Lorigan P, et al. PD-L1 expression as a potential predictive biomarker. The Lancet Oncology. 2015;16(13):1285–7. Epub 2015/10/05. doi: 10.1016/S1470-2045(15)00307-1 . 26433815

[39] Chan TA, Yarchoan M, Jaffee E, Swanton C, Quezada SA, Stenzinger A, et al. Development of tumor mutation burden as an immunotherapy biomarker: utility for the oncology clinic. Annals of oncology: official journal of the European Society for Medical Oncology. 2019;30(1):44–56. Epub 2018/11/06. doi: 10.1093/annonc/mdy495 . 30395155 PMC6336005

[40] Dudley JC, Lin MT, Le DT, Eshleman JR. Microsatellite Instability as a Biomarker for PD-1 Blockade. Clinical cancer research: an official journal of the American Association for Cancer Research. 2016;22(4):813–20. Epub 2016/02/18. doi: 10.1158/1078-0432.CCR-15-1678 . 26880610

[41] Motzer RJ, Escudier B, McDermott DF, George S, Hammers HJ, Srinivas S, et al. Nivolumab versus Everolimus in Advanced Renal-Cell Carcinoma. The New England journal of medicine. 2015;373(19):1803–13. Epub 2015/09/26. doi: 10.1056/NEJMoa1510665 . 26406148 PMC5719487

[42] Diakos CI, Charles KA, McMillan DC, Clarke SJ. Cancer-related inflammation and treatment effectiveness. The Lancet Oncology. 2014;15(11):e493–503. Epub 2014/10/05. doi: 10.1016/S1470-2045(14)70263-3 . 25281468

[43] Elinav E, Nowarski R, Thaiss CA, Hu B, Jin C, Flavell RA. Inflammation-induced cancer: crosstalk between tumours, immune cells and microorganisms. Nature reviews Cancer. 2013;13(11):759–71. Epub 2013/10/25. doi: 10.1038/nrc3611 . 24154716

[44] Zhang H, Gao L, Zhang B, Zhang L, Wang C. Prognostic value of platelet to lymphocyte ratio in non-small cell lung cancer: a systematic review and meta-analysis. Sci Rep. 2016;6:22618. Epub 2016/03/05. doi: 10.1038/srep22618 . 26939789 PMC4778054

[45] Qiang G, Liang C, Xiao F, Yu Q, Wen H, Song Z, et al. Prognostic significance of platelet-to-lymphocyte ratio in non-small-cell lung cancer: a meta-analysis. OncoTargets and therapy. 2016;9:869–76. Epub 2016/03/10. doi: 10.2147/OTT.S96804 . 26955285 PMC4768894

[46] Lefrançais E, Ortiz-Muñoz G, Caudrillier A, Mallavia B, Liu F, Sayah DM, et al. The lung is a site of platelet biogenesis and a reservoir for haematopoietic progenitors. Nature. 2017;544(7648):105–9. Epub 2017/03/23. doi: 10.1038/nature21706 . 28329764 PMC5663284

[47] Tesselaar ME, Osanto S. Risk of venous thromboembolism in lung cancer. Current opinion in pulmonary medicine. 2007;13(5):362–7. Epub 2007/10/18. doi: 10.1097/MCP.0b013e328209413c . 17940477

[48] Rodriguez-Martinez A, Simon-Saez I, Perales S, Garrido-Navas C, Russo A, de Miguel-Perez D, et al. Exchange of cellular components between platelets and tumor cells: impact on tumor cells behavior. Theranostics. 2022;12(5):2150–61. doi: 10.7150/thno.64252 35265204 PMC8899588

[49] In ’t Veld S, Wurdinger T. Tumor-educated platelets. Blood. 2019;133(22):2359–64. Epub 2019/03/06. doi: 10.1182/blood-2018-12-852830 . 30833413

[50] Best MG, Wesseling P, Wurdinger T. Tumor-Educated Platelets as a Noninvasive Biomarker Source for Cancer Detection and Progression Monitoring. Cancer research. 2018;78(13):3407–12. Epub 2018/06/21. doi: 10.1158/0008-5472.CAN-18-0887 . 29921699

[51] Xu H, He A, Liu A, Tong W, Cao D. Evaluation of the prognostic role of platelet-lymphocyte ratio in cancer patients treated with immune checkpoint inhibitors: A systematic review and meta-analysis. Int Immunopharmacol. 2019;77:105957. Epub 2019/11/05. doi: 10.1016/j.intimp.2019.105957 . 31677498

[52] Zhang N, Jiang J, Tang S, Sun G. Predictive value of neutrophil-lymphocyte ratio and platelet-lymphocyte ratio in non-small cell lung cancer patients treated with immune checkpoint inhibitors: A meta-analysis. Int Immunopharmacol. 2020;85:106677. Epub 2020/06/13. doi: 10.1016/j.intimp.2020.106677 . 32531712

