# Supplementary figures and images for: Prognostic value of the platelet-to-lymphocyte ratio in lung cancer patients receiving immunotherapy: A systematic review and meta-analysis
Source: PLoS One. 2022 May 6;17(5):e0268288. doi: 10.1371/journal.pone.0268288 (PMC9075650; doi:10.1371/journal.pone.0268288)

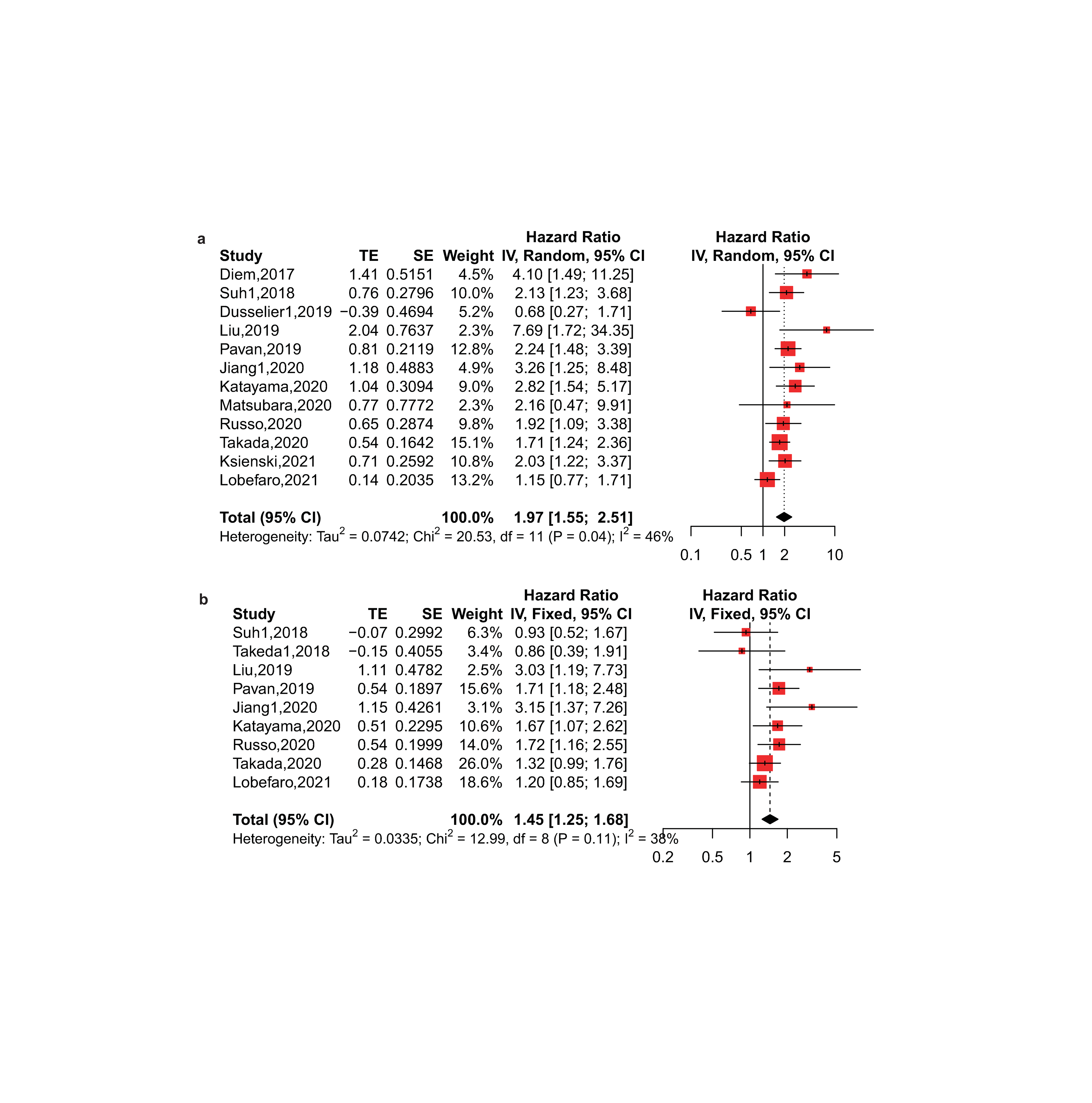

Supplement: S1 Fig — a) forest plot for OS; b) forest plot for PFS. (TIF) [file pone.0268288.s002.tif]
