# Supplementary material for: Prognostic value of the platelet-to-lymphocyte ratio in lung cancer patients receiving immunotherapy: A systematic review and meta-analysis
Source: PLoS One. 2022 May 6;17(5):e0268288. doi: 10.1371/journal.pone.0268288 (PMC9075650; doi:10.1371/journal.pone.0268288)
Supplement: S1 File — (DOCX) [file pone.0268288.s003.docx]

**Supplementary File 1**

**Search strategy for meta-analysis (PubMed via NLM)**

Search terms: platelet-to-lymphocyte ratio and lung cancer patients receiving immunotherapy

Population: lung cancer patients receiving immunotherapy

#1: lung neoplasms[MeSH] OR Pulmonary Neoplasms OR Neoplasms, Lung OR Lung Neoplasm OR Neoplasm, Lung OR Neoplasms, Pulmonary OR Neoplasm, Pulmonary OR Pulmonary Neoplasm OR Lung Cancer OR Cancer, Lung OR Cancers, Lung OR Lung Cancers OR Pulmonary Cancer OR Cancer, Pulmonary OR Cancers, Pulmonary OR Pulmonary Cancers OR Cancer of the Lung OR Cancer of Lung OR Adenocarcinoma of Lung[MeSH]

#2: immunotherapy OR immune checkpoint inhibitor OR programmed death 1 OR PD-1 OR programmed death ligand 1 OR PD-L1 OR cytotoxic T-lymphocyte-associated protein 4 OR CTLA-4 OR nivolumab OR pembrolizumab OR atezolizumab OR avelumab OR durvalumab OR ipilimumab OR tremelimumab

#3: #1 AND #2

Exposure: platelet-to-lymphocyte ratio

#4: platelet-to-lymphocyte ratio OR platelet lymphocyte ratio OR PLR OR lymphocyte-to-platelet ratio OR lymphocyte platelet ratio OR LPR

Combined sets:

#3 AND #4
